# Improved artificial origins for phage Φ29 terminal protein-primed replication. Insights into early replication events

**DOI:** 10.1093/nar/gku660

**Published:** 2014-07-31

**Authors:** Pablo Gella, Margarita Salas, Mario Mencía

**Affiliations:** Centro de Biología Molecular “Severo Ochoa” (Consejo Superior de Investigaciones Científicas – Universidad Autónoma de Madrid), Universidad Autónoma, Cantoblanco, 28049 Madrid, Spain

## Abstract

The replication machinery of bacteriophage Φ29 is a paradigm for protein-primed replication and it holds great potential for applied purposes. To better understand the early replication events and to find improved origins for DNA amplification based on the Φ29 system, we have studied the end-structure of a double-stranded DNA replication origin. We have observed that the strength of the origin is determined by a combination of factors. The strongest origin (30-fold respect to wt) has the sequence CCC at the 3′ end of the template strand, AAA at the 5′ end of the non-template strand and 6 nucleotides as optimal unpairing at the end of the origin. We also show that the presence of a correctly positioned displaced strand is important because origins with 5′ or 3′ ssDNA regions have very low activity. Most of the effect of the improved origins takes place at the passage between the terminal protein-primed and the DNA-primed modes of replication by the DNA polymerase suggesting the existence of a thermodynamic barrier at that point. We suggest that the template and non-template strands of the origin and the TP/DNA polymerase complex form series of interactions that control the critical start of terminal protein-primed replication.

## INTRODUCTION

DNA linear elements containing proteins covalently linked at their 5′ ends have been reported or proposed to exist in all domains of life (1,2,3,4). These DNA elements are typically viruses or plasmids (5). The proteins attached to the DNA elements, called terminal proteins (TPs), in most cases act together with special DNA polymerases, encoded also by these genetic elements, in the task of replicating these DNAs (1). In general, the DNA replication role of the TPs is to substitute for DNA or RNA primers when the DNA polymerases replicate the 3′ DNA ends of these linear elements. In this way, the problems associated with the replication of the ends of a linear DNA are solved (5).

In this type of genomes, the ends of the DNA constitute true replication origins, and they are characterized, apart from having TPs covalently linked at the 5′ ends, by having, in most cases, at both ends of the corresponding genome, inverted terminal repeat (ITR) sequences (1). The ITRs have been reported to be recognized by the complexes formed by TPs and DNA polymerases plus other DNA binding proteins in the events that lead to the initiation of TP-primed replication (1,6,7,8). Thus, for instance, in the case of Adenovirus, the ITRs are about 100 bp long and they have been shown to interact with the adenovirus TP, the DNA polymerase and the double-stranded DNA binding protein, as well as with the cellular factors NFI and NFIII, and all these proteins are required for full effectiveness of the replication origin (6). In the case of the *Streptomyces* linear plasmids or chromosomes, the ITRs are proposed to be bound by the TP and by another DNA binding protein called Tap (8,9 see also 10). In some cases the TP-primed DNA replication is merely a telomere-like mechanism to maintain the genome ends and there are regular host-type replication origins that serve to replicate the majority of the genome, like, for example, in some *Streptomyces* plasmids (11,12). In many other cases there are no other origins apart from the ends of the genome, and the TP-primed machinery replicates the full length of the DNA (5).

The replication machinery of the *Bacillus subtilis* bacteriophage Φ29 is the TP-primed replication system best characterized *in vitro* by biochemical studies using purified proteins (13). In this system, the genome ends are short ITRs of 6 bp with the sequence ^5′^AAAGTA^3′^ (14,15). A minimal set of proteins has been defined for efficient *in vitro* amplification of the TP-containing 19 Kb phage genome (16). These phage-encoded proteins are the TP, the DNA polymerase, the single-stranded DNA binding protein p5 (SSB) and the double-stranded DNA binding protein p6. According to the current model, the p6 would be the first protein to bind to the genome ends. The p6 binding is mediated by the recognition of a 24 bp phased bendability pattern of the DNA that generates a right-handed superhelical complex with DNA regions up to 200 bp from the ends (17, see 18 for a review). The binding of p6 is proposed to act both by favoring the unpairing of the DNA ends (19) and also by recruiting the complex formed by the TP and the DNA polymerase to the origin (20,21). As mentioned above, the replication origins also have the parental TP bound covalently to the 5′ ends of the DNA and this TP molecule strongly enhances the initiation step of Φ29 DNA replication (22). Some of the last 12 bp of the DNA ends, that includes the 6 bp ITR at the very extreme, have been shown to be important for the initiation of replication (23).

When the Φ29 TP/DNA polymerase heterodimer binds to the origin, the 3′ end of the DNA is proposed to occupy the template strand position in the DNA polymerase structure and then the second T from the 3′ end serves as template for the addition of dAMP to the OH group of Ser232 of the TP (24). This implies that the TP effectively mimics the presence of a DNA primer at the double-stranded DNA binding cleft of the DNA polymerase, and the role of the free 3′ OH group of a DNA primer is taken by the OH group of Ser232 of the TP (25). Once the second position of the template has been used, the TP-dAMP complex is displaced back one position and the dAMP bound to the TP pairs with the first position of the template (T), and the second position (T) acts again as a template for the addition of the second dAMP to the 3′ OH of the TP-linked dAMP. This mechanism, involving the repositioning of the TP-dAMP complex on the template is called sliding-back (25). The sliding-back mechanism was first demonstrated for the phage Φ29 DNA replication, and, since then, several variants of the mechanism have been reported for TP-containing DNA elements (26,27,28,29). This type of mechanism requires the presence of short sequence repetitions at the DNA ends, like the repetition ^5′^AAA^3′^ in the case of Φ29. Apart from the critical importance of the first and second positions, the presence of T also at the third position of the template strand has been shown to improve the efficiency of the initiation of replication (24,30).

After the initiation phase takes place, the DNA polymerase continues with the synthesis of the nascent DNA strand, still forming a complex with the TP until the newly produced DNA is 6 to 9 nucleotides long during a stage called ‘transition’. After the transition, the TP and DNA polymerase dissociate from each other (31) and the enzyme replicates the 19 Kb genome of the phage as a standard DNA dependent-DNA polymerase. The DNA polymerase is endowed with very potent strand displacement capacity and high processivity and does not require additional factors like helicases or sliding clamps. Both ends of the phage DNA are utilized as origins and the genome duplication is achieved in a process of symmetrical replication (32).

The crystallographic structure of the TP/DNA polymerase heterodimer has been determined (25) and it shows how the TP is divided into three structural domains: (i) the C-terminal domain (Ct; residues 171–266), which contains the Ser232 priming residue, (ii) the intermediate domain (I; amino acids 73–170) that contributes to the surface of interaction TP/DNA polymerase especially with the region TPR1 of the polymerase and (iii) the N-terminal domain (Nt; residues 1–72) whose tertiary structure was not solved in the crystal. The Nt domain has non-sequence-specific DNA binding capacity, and is partially dispensable for *in vitro* DNA replication, since a TP lacking the Nt domain does not initiate from templates without parental TP and it requires p6 for the efficient replication of the phage genome (21,33).

As mentioned above, efficient amplification of the TP-containing Φ29 genome has been obtained using the four essential replication proteins, reaching amplification factors of 1000-fold (16). Based on this system, we have developed a method to obtain *in vitro* TP-primed amplification of heterologous DNAs that contain the Φ29 replication origins but do not have TP covalently linked beforehand (34). The fact that the product of amplification is DNA covalently linked to a TP makes the method a useful platform for novel technologies, such as DNA display, or *in vitro* evolution of proteins by linking phenotype to genotype (35), given that new features can be added to the TP by fusion of protein domains with selectable functions that can be coded for by the linked DNA molecule.

In order to gain knowledge on the recognition of the Φ29 DNA replication origins by the TP/DNA polymerase heterodimer and to try to increase the efficiency of the first stages of the replication process for possible applications, we have performed a study on the efficiency of origin utilization by the TP/DNA polymerase complex using different combinations of origin sequences and end-structures. The study resulted in the finding of novel origins more efficient than the wild-type ones, and it also sheds light on the early characteristic events of TP-primed DNA replication.

## MATERIALS AND METHODS

### Nucleotides and DNAs

Unlabeled nucleotides and dideoxynucleotides were purchased from General Electric Healthcare. [α-^32^P]dATP (3000 Ci/mmol), [α-^32^P]dGTP and [α-^32^P]dCTP (3000 Ci/mmol) were supplied by PerkinElmer Inc. Oligonucleotides were obtained from Sigma-Aldrich and PAGE purified. TP-containing Φ29 DNA (Φ29 TP-DNA) was obtained as described (36). The oligonucleotide hybridizations to obtain the different dsDNA origins were performed in TE buffer (10 mM Tris–HCl, pH 7.5, 1 mM ethylenediaminetetraacetic acid (EDTA)) at 8 μM each oligonucleotide in a Biorad (Hercules, CA, USA) MyCycler thermal cycler using the following program, 95°C for 5 min, 93°C for 30 s, 85°C for 10 min, 80°C for 10 min, 75°C for 5 min, 70°C for 5 min, 65°C for 5 min, 45°C for 60 min and then holding at 4°C.

### Proteins

DNA polymerase (37), TP and protein p6 (34) were purified as described. TP mutant TPΔNt, which lacks the Nt domain, was purified as described (21).

### Replication assay

The incubation mixture contained, in 25 μl, buffer MRI [50 mM Tris–HCl, pH 7.5, 10 mM MgCl_2_, 5% (v/v) glycerol, 1 mM DTT, 0.1 mg/ml bovine serum albumin] plus ammonium sulphate to 20 mM final concentration, 5 μM each dATP, dGTP, dTTP, dCTP and (1 μCi) [α-^32^P]dATP, 625.5 pM of Φ29 TP–DNA or 313 nM of the specified double-stranded oligonucleotide, 150 nM of TP, 30 nM of wild-type Φ29 DNA polymerase and incubated for 5 min at 30°C. The reactions were stopped by adding 10 mM EDTA and 0.1% sodium dodecyl sulphate (SDS), final concentrations. Then, the samples were ﬁltered through Sephadex G-50 spin columns in the presence of 0.1% SDS, analyzed by SDS-polyacrylamide gel electrophoresis (PAGE), as described (36), and detected by autoradiography. Quantitation of gel bands was performed on a BAS1000 phosphorimager equipment. Averages and standard deviations are presented from at least three independent experiments. The number of labeled nucleotides incorporated per replicated DNA molecule was taken into account for the calculations in all the experiments. The amount of DNA product for each origin was normalized considering the DNA produced by the wt origin as 1. A protein-primed replication assay carried on Φ29 TP–DNA was used as molecular weight marker and additional standardization reference in all experiments. The rest of the conditions were essentially the standard ones utilized in previous studies.

### TP-dAMP formation (protein-primed initiation assay)

The incubation mixture contained, in 25 μl, buffer MRI plus ammonium sulphate to 20 mM final concentration, 0.1 μM and 1 μCi of the corresponding dNTP and [α-^32^P] dNTP, respectively, 313 nM of the specified double-stranded oligonucleotide, 150 nM of puriﬁed TP and 30 nM of wild-type DNA polymerase, and incubated for 5 min at 30°C. To test its effect on 68mer double-stranded oligonucleotides 10.5 μg Φ29 p6 was added when indicated. The reactions were stopped by adding 10 mM EDTA and 0.1% SDS, final concentrations. Then, samples were processed and analyzed as described above.

### Transition assay

The incubation mixture contained, in 25 μl, buffer MRI plus ammonium sulphate to 20 mM final concentration, 313 nM of the specified double-stranded oligonucleotide, 150 nM of TP and 30 nM of wild-type DNA polymerase, and incubated for 5 min at 30°C. A mixture of different deoxynucleotides and dideoxynucleotides were added to the reaction in order to produce sequential stops to analyze the process of transition: for wild-type double-stranded oligonucleotide were added dATP and ddGTP, dATP or dGTP and ddTTP, dATP, dGTP or dTTP and ddCTP (dNTPs:ddNTPs = 5:100 μM) or 5 μM dNTPs when indicated plus 1 μCi of [α-^32^P]dATP; for A/C and 6m double-stranded oligonucleotides were added dGTP and ddTTP, dGTP or dTTP and ddATP, dATP, dGTP or dTTP and ddCTP (dNTPs:ddNTPs = 5:100 μM) or 5 μM dNTPs when indicated plus 1 μCi of [α-^32^P]dGTP. The reactions were stopped by adding 10 mM EDTA and 0.1% SDS, final concentrations. Then, samples were ﬁltered through Sephadex G-50 spin columns in the presence of 0.1% SDS and analyzed by electrophoresis in SDS-12% polyacrylamide gels (dimensions, 360 × 280 × 0.5 mm) to obtain enough resolution to distinguish the TP bound to all the elongation products. The gel bands were detected by autoradiography and the quantitation was performed on a BAS1000 phosphorimager equipment.

## RESULTS

### Effect of group substitutions and unpairing of the 3 first nucleotides on replication origin utilization efficiency

As mentioned in the Introduction, an efficient sliding-back mechanism operates optimally on the ^3′^TTT^5′^ nucleotide repeat at the template strand end. Since there was little information on the effect of the first three nucleotides identity on the potency of Φ29 dsDNA origins, we mutated those positions as homooligomeric blocks of three nucleotides at both template and non-template (displaced) strands in all possible combinations. This approach would allow us to test the effects of the sequence, and also the end unpairing in those cases where the combination of the oligonucleotides produces non-complementary ends. To simulate a DNA end of the Φ29 genome lacking TP, we designed 29-mer oligonucleotides that were hybridized to generate the dsDNA ends (see Supplementary Table SI for oligonucleotide sequences and origin combinations). The labeled nucleotide was dATP for quantitative comparison of full-length products, and as a consequence the initiation positions would not be labeled for templates other than those with ^3′^TTT^5′^ at the 3′ end.

**Figure 1.**
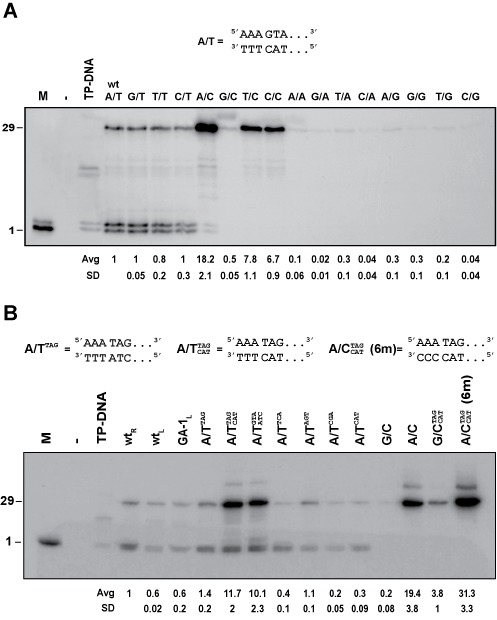
(A) Effect of group substitution of the first three nucleotides at the template and displaced strands on replication origin utilization. Double-stranded DNA origins were assembled with synthetic oligonucleotides to generate the different end combinations. Reactions were performed as described in Materials and Methods using [α^32^P] dATP as labeling nucleotide, and the reaction products were analyzed by high-resolution SDS-PAGE with 12% acrylamide. The positions corresponding to TP-dAMP (1) and full length product (TP-29 nucleotides ssDNA) are indicated. M, initiation reaction marker (reaction using TP-DNA as template and dATP as the only nucleotide, see Materials and Methods section); TP-DNA, control reaction with TP-containing Φ29 genome as template (this reaction produces the initiation and partial elongation bands [+15,+16] in addition to full-length Φ29 DNA [not shown]). The different pairs of oligonucleotides hybridized to generate the artificial origins are noted as follows (see scheme above Figure 1): the first letter stands for the first three nucleotides of the displaced strand and the second letter stands for the first three nucleotides of the template strand (for example, A/T denotes an origin with the paired sequence at the end AAA/TTT). The rest of the origin is the sequence of the Φ29 DNA right end from positions 4–29 (see Materials and Methods section). For example, the wt origin is the A/T combination. At the bottom of the figure are shown the mean values of the quantification of the intensities of the bands corresponding to full length products compared to the wt (wt = 1), with their corresponding standard deviations. Values are the average of at least three independent experiments. (B) Effect of nucleotide substitutions at positions 4–6 and unpairings on replication origin utilization. As previously, double-stranded DNAs were assembled with synthetic oligonucleotides to generate the different combinations. Reactions were analyzed by 12% SDS-PAGE. The positions corresponding to TP-dAMP (1) and full length product (TP-29 nucleotides ssDNA) are indicated. wt_R_, Φ29 DNA right origin of replication (29 bp); wt_L_, Φ29 DNA left origin of replication (29 bp), GA-1_L_, GA-1 left origin of replication (29 bp); as in Figure 1, two capital letters indicate the first three nucleotides of the displaced strand and the first three nucleotides of the template strand, respectively; in addition to that, superindexes alone (A/T^NNN^) denote the sequences from positions 4–6 of the displaced strand if other than wt and paired. Superindexes and subindexes (${\rm A/T}_{{\rm CAT}}^{{\rm TAG}}$) denote the sequences from positions 4–6 of the displaced and template strands, respectively, when they are unpaired (see scheme above Figure 1B).

First, we checked with appropriate markers that the size of the product DNA linked to the TP was the correct one, this is 29 nucleotides long (see Supplementary Figure S1). We also checked that the level of initiation or replication produced from the non-origin DNA end was negligible (not shown). The results (Figure 1A) showed that the level of full-length oligomer replication is very low for template strands that have purines at the 3′ end (N/A or N/G, being N any nucleotide), intermediate for wild-type templates (N/T) and highest for C-templates (N/C) with the exception of the pairing G/C. Regarding the non-template strand, there are no significative differences for any of the combinations with the wild-type template strand, whether they are paired or not. However, for the CCC-ended templates we observe the highest activity for the combination with AAA at the displaced strand (lane A/C, 18-fold respect to the wild-type), a somewhat lower activity but still 7-fold over the wild-type for origins with TTT or CCC at the displaced strand (lanes T/C and C/C), and a comparatively low activity (0.5 times the wild-type) for the G/C paired origin. This suggests that there is a negative effect for the pairing G/C, and a clear positive effect for the unpairings C/C, T/C and especially A/C. In the case of the A/C template there are two weak initiation bands even when the labeled nucleotide is dATP, not dGTP; we consider these bands as errors of incorporation since the total level of TP-DNA product is particularly high for this template.

From these results we can conclude that, for origin utilization, it is important the identity of the first-triplet at the template strand with a clear preference for Cs followed by Ts, and, in the case of C-templates, we observe a favorable effect of the DNA unpairing and a clear preference for A at the first positions of the displaced strand.

### Effect of substitutions and unpairing of nucleotides 4–6 on replication origin utilization efficiency

Once the effect of block substitutions of the first three nucleotides of the origin was determined, the effect of substitutions and unpairing at positions 4–6 was tested. We started by comparing the activities of the Φ29 right and left ends, that have the same nucleotide sequence up to position 6 due to the identical 6bp-ITRs, but differ from position 7 onwards (see Supplementary Table SI), with the observation that the right end was a little stronger as replication origin than the left end (Figure 1B, compare lanes wt_R_ and wt_L_). This suggests that the sequence from position 7 onwards does have an effect on the strength of the origin. On the other hand, analysis of positions 4–6 was carried out by sequence comparison with phage GA-1. Phage GA-1 belongs to the same family as Φ29 but it is sufficiently different so that its ITR nucleotide sequence from positions 4–6 is ^5′^TAG^3′^ while that of Φ29 is ^5′^GTA^3′^ (28). We tested an origin composed by the first 12 nucleotides of GA-1 left end and the rest of Φ29 right end sequence resulting in an activity similar to that of the wt_L_ (lane GA-1_L_). Then, nucleotides from positions 4–6 of GA-1 were introduced to substitute for the same positions in the Φ29 right end and the activity obtained was similar to that of the wt_R_ (lane A/T^TAG^). This suggests that positions 4–6 of the Φ29 origin (^5′^GTA^3′^) can be changed to ^5′^TAG^3′^ without a negative effect. Next, we tested four different random sequences at positions 4–6 and observed that one (^5′^AGT^3′^, lane A/T^AGT^) reached wild-type level, while three of them (TCA, CGA, CAT (5′ to 3′)) gave lower activity. Altogether, the results suggest that some changes at positions 4–6 are permitted.

Given that origins having the sequence ^5′^TAG^3′^ at positions 4–6 produced an activity similar to that of the wild type, we then tested the effect of the unpairing at positions 4–6 by hybridizing the corresponding oligonucleotides, generating the two possible combinations (AAATAG/TTTCAT and AAAGTA/TTTATC). Interestingly, both combinations gave activities around 10-fold higher than that of the wild-type (lanes ${\rm A/T}_{{\rm CAT}}^{{\rm TAG}}$ and ${\rm A/T}_{{\rm ATC}}^{{\rm GTA}}$), being the first combination slightly stronger. This suggests that unpairing of positions 4–6 strongly stimulates origin utilization. Taking into account the results from the previous section, in which the A/C unpaired end was the strongest origin (lane A/C of Figure 1A), the segment (AAA/CCC) was combined with the 4–6 also unpaired segment TAG/CAT. As shown in Figure 1B, the template with unpairings from position 1–6 produced the strongest activity obtained so far, having 30-fold the wild-type activity (lane ${\rm A/C}_{{\rm CAT}}^{{\rm TAG}}$). If we change the upper band AAA to GGG, so it forms pair with the CCC template, the activity is reduced to 3.8-fold of the wild-type activity (lane ${\rm G/C}_{{\rm CAT}}^{{\rm TAG}}$). All this indicates that some unpairings can be combined in such a way that their positive effects on origin utilization are additive. The results suggest that up to position 6 the pairing of the bases poses a barrier to origin utilization that can be alleviated by artificial unpairing as in the artificial origins.

Given that the AAATAG/CCCCAT origin, having six mismatches (6m), had the strongest activity observed so far, we decided to explore sequence variations around this combination but keeping the six nucleotides unpairing. The results shown in Table 1 indicate that there is some effect of the sequence from positions 4–6 in both strands, this is within the six mismatch-context. The observed activities range from 27% to 109%, considering the 6m activity as 100%. Also, the constructs not having AAA at the 5′ end still keep 65% (TTT at the 5′) or 71% (the randomly chosen sequence ^5′^CTCACG^3′^) of the 6m activity. These results suggest that the sequence from positions 4–6 plays a role in the activity of the six mismatched origins.

**Table 1.** Sequence effect on the six-mismatch unpaired origins. Different end-structures were generated by hybridization of the corresponding oligonucleotides. The sequences of the first column correspond to the 5′ and 3′ six nucleotides at the termini of the displaced and template strands of the origins, respectively. The second column (Avg%) shows the utilization of the different origins as percentage respect to the 6m construct value to facilitate comparison. The percentage is the average of a minimum of three independent experiments. In the third column are listed the standard deviations corresponding to the averages in the second column.

		Avg (%)	SD
(wt)	AAAGTA	3	1.2
	TTTCAT		
(G/C)	GGGGTA	0.7	0.1
	CCCCAT		
(A/C)	AAAGTA	49.2	12
	CCCCAT		
(6m)	AAATAG	100	
	CCCCAT		
	AAACAT	109.5	17.8
	CCCCAT		
	AAACGA	66.3	10.5
	CCCATC		
	AAAGTA	58.1	4.1
	CCCATC		
	AAAAGT	57.1	7.5
	CCCATC		
	AAATAG	49.1	3.3
	CCCGCT		
	AAACGA	43.8	6.3
	CCCGTA		
	AAAGTA	43.1	3.9
	CCCGTA		
	AAATCA	27.6	6
	CCCGTA		
	TTTTAG	64.9	14.1
	CCCCAT		
	CTCACG	71.1	12.3
	CCCCAT		

### Effect of 5′ overhangs on the strength of the replication origin

**Figure 2.**
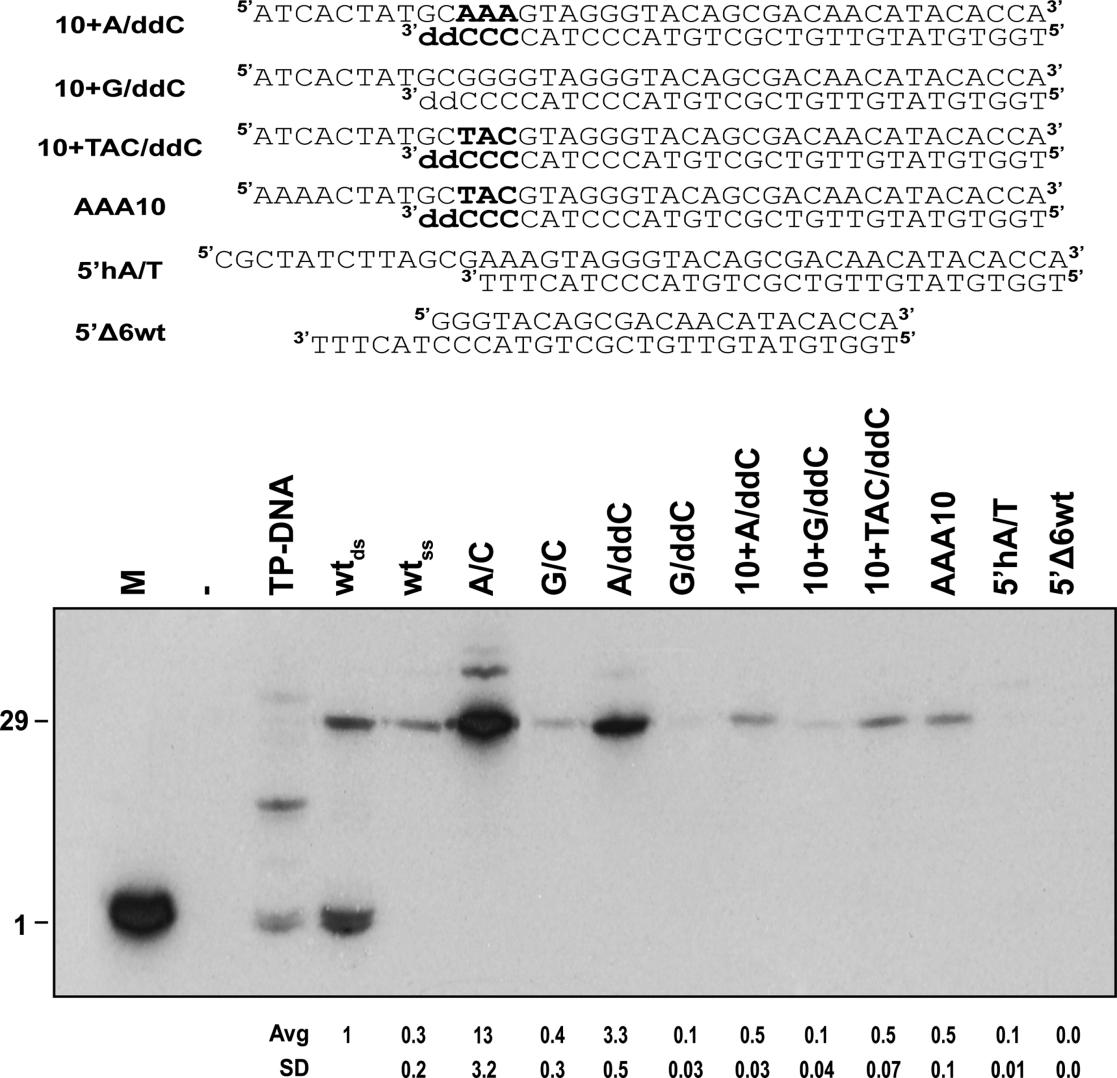
Effect of 5′ overhangs on the strength of the replication origin. Different end-structures were generated by hybridization of the corresponding oligonucleotides. wt_ds_, Φ29 DNA right origin of replication (29 bp); wt_ss_, Φ29 DNA right origin of replication, only template strand (29 nucleotides); A/ddC and G/ddC follow the same nomenclature as in Figure 1 but the first nucleotide at the template strand 3′ position is dideoxyCMP; 10+A/ddC, indicates an addition of 10 random nucleotides (sequence ATCACTATGC) at the 5′ position of the A/ddC origin; 10+G/ddC, the same addition on the G/ddC origin; 10+TAC/ddC, the same addition on a TAC/ddCCC ended origin; AAA10, sequence AAACTATGC added at the 5′ end of 10+TAC/ddC; 5′hA/T, an addition of 13 nucleotides at the 5′ end forming a self-complementary 5 bp hairpin on the wt origin (^5′^**CGCTA**TCT**TAGCG**^3′^, in bold the self-complementary sequence); 5′Δ6wt, deletion of the first six nucleotides at the 5′ end of the displaced strand on the wt origin (see Supplementary Table S1 for oligonucleotides sequences and origin structures).

From previous work it was known that the replication machinery can utilize ends with 3′ protrusions 5-nucleotides long, because the intrinsic 3′ to 5′ exonuclease activity of the Φ29 DNA polymerase can eliminate the extensions to generate the wild-type, blunt ends (34). Since the unpairing of the ends has a positive effect on origin utilization, we tested the effect of an extended displaced strand on the origin strength with the idea that an increased number of interactions between a non-template protruding single strand and the TP/DNA polymerase heterodimer could stabilize the initiation complex and produce higher origin utilization. Thus, we tested origins with different 5′ 10-nucleotides ssDNA overhangs at the displaced strand, taking into account that these overhangs cannot be degraded by the 3′-5′ exonuclease activity of the Φ29 DNA polymerase. The overhangs were 10-nucleotides long in order to provide enough ssDNA to cover a substantial portion of the TP/DNA polymerase heterodimer. Since a 3′-recessive DNA end would be a good primer for the standard polymerization activity, and this would alter the ends to analyze, we constructed non-extensible oligonucleotides with dideoxy-CMP (ddC) as the last nucleotide at the 3′ position of the template strand. As a control for the possible additional effect of the templates with ddC, we tested the two constructions A/C and G/C with and without ddC. In Figure 2 we observe that both A/C and G/C origins with ddC (lanes A/ddC and G/ddC, respectively) at the very end have 25% of the activity of their homologous origins with dC at the end. Although the reason for this is unknown, a possibility is that the 3′ hydroxyl group may be important in the interaction with the TP at the early event of initiation. As an additional fact, we also observed that the replication activity from a double-stranded origin (wt_ds_) was stronger than that of a single-stranded origin (wt_ss_), having just the template strand. This stresses the importance of the displaced strand in the early process of replication. Regarding the 5′-overhang origins we observed that they all lead to markedly lower activities than the A/ddC non-overhang control. It happens so if the origin has the 5′ protruding sequence ATCACTATGC and then AAA/ddCCC, or the same 5′ extension and then TAC/ddCCC (lanes 10+A/ddC and 10+TAC/ddC), or even if it has the 5′-overhang sequence AAACTATGC and then TAC/ddCCC (lane AAA10); in all cases the activity was lower than that of the A/ddC and the wild-type origins. As expected, the overhang with G/C pairing at the end of the double-stranded section showed an activity even lower than that of the other origins (lane 10+G/ddC). Also, a wild-type origin with a 5′-overhang that forms a self-complementary hairpin (lane 5′hA/T) and other modified wild-type origin lacking six nucleotides of the displaced strand (lane 5′Δ6wt) showed essentially no activity, probably because the heterodimer has to face the opening of a G/C paired double-stranded DNA at the same time as initiation is taking place. These results point to the importance of the displaced strand in the process of origin utilization, and they also suggest that, when the displaced strand is made artificially longer than the template, it does not produce stimulation, contrary to what happens when we simulate the effect of an origin being opened by using unpaired oligonucleotides of the same length.

### Preference of the TP/DNA polymerase heterodimer for the sequence AAA at the 5′ end of the displaced strand

**Figure 3.**
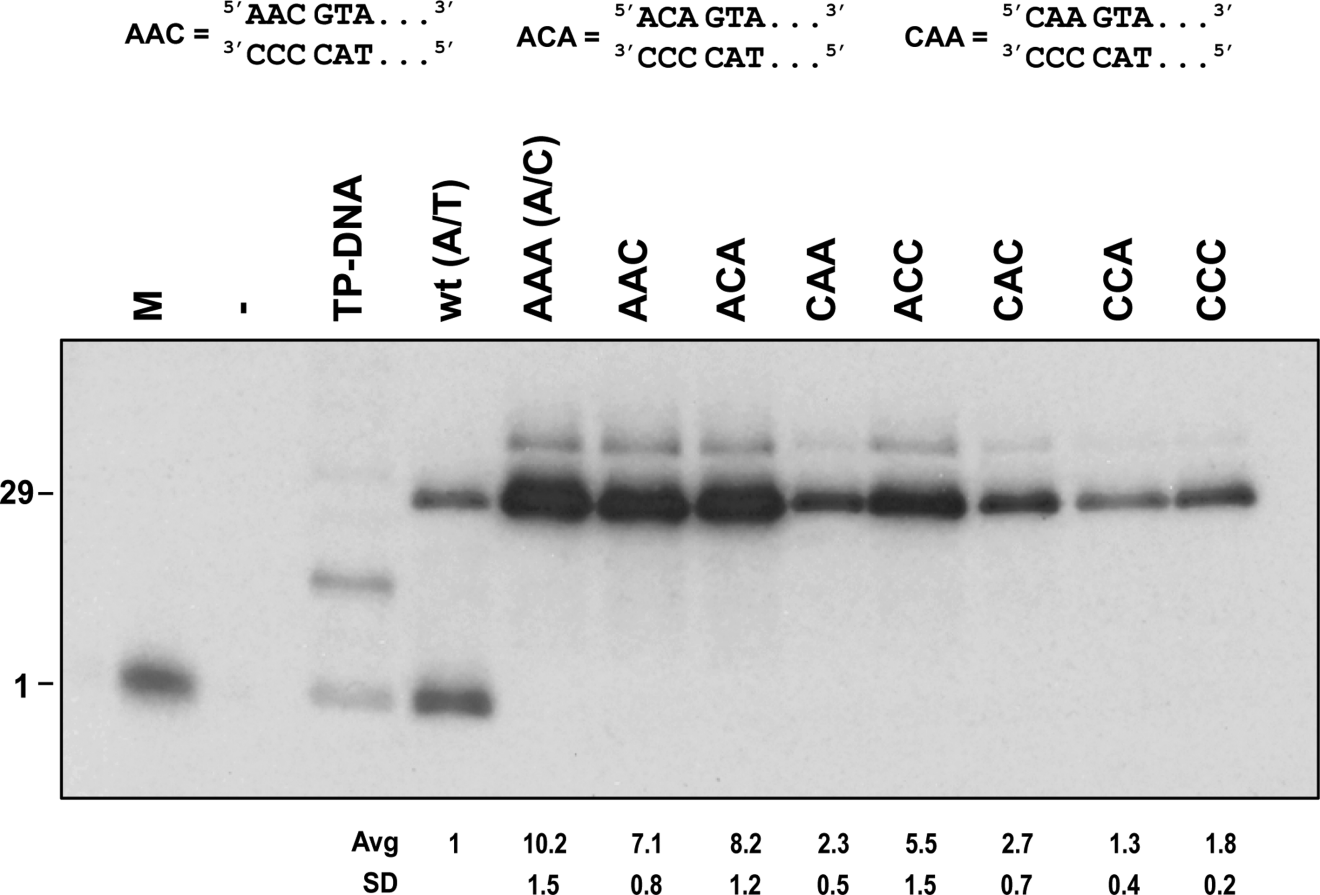
Preference of the TP/DNA polymerase heterodimer for AAA at the 5′ end of the displaced strand. Notation as before for wt and A/C. The A/C construct is the basis for all the modifications. NNN indicates the last three nucleotides of the 5′ end of the displaced strand, the template strand being ^3′^CCC^5′^ in all cases.

The best activities so far were obtained with origins with the unpairing A/C at the end. For that reason, we decided to characterize the observed preference for the sequence AAA at the 5′ end of the displaced strand. We started from the original 3-mismatched A/C origin (A/C = AAA in Figure 3), because it shows a clearer effect of the AAA sequence than other origins. We substituted all As at the displaced strand individually or in combinations with Cs. The results (Figure 3) showed that there is a strong preference for A at the first position from the 5′ end (compare lane AAA with CAA). The substitution of the second A also reduces the activity of the origin (lanes AAA versus ACA and lanes CAC versus CCC), although the effect is lower than that of the first A. However, for position 3 there are cases where its substitution reduces the activity (lanes AAA versus AAC and lanes ACA versus ACC) and other cases where the change increases slightly the activity of the origin (lanes CAA versus CAC and CCA versus CCC). Comparing the origins with just one A (ACC, CAC and CCA) a possible ranking of importance can be suggested, first A better than second A and this is better than the third A. The results suggest that there is a preference for AAA at the 5′ end of the displaced strand by the TP/DNA polymerase complex and that the first A is the most important one for that preference.

### Optimal number of unpaired nucleotides at the replication origin

**Figure 4.**
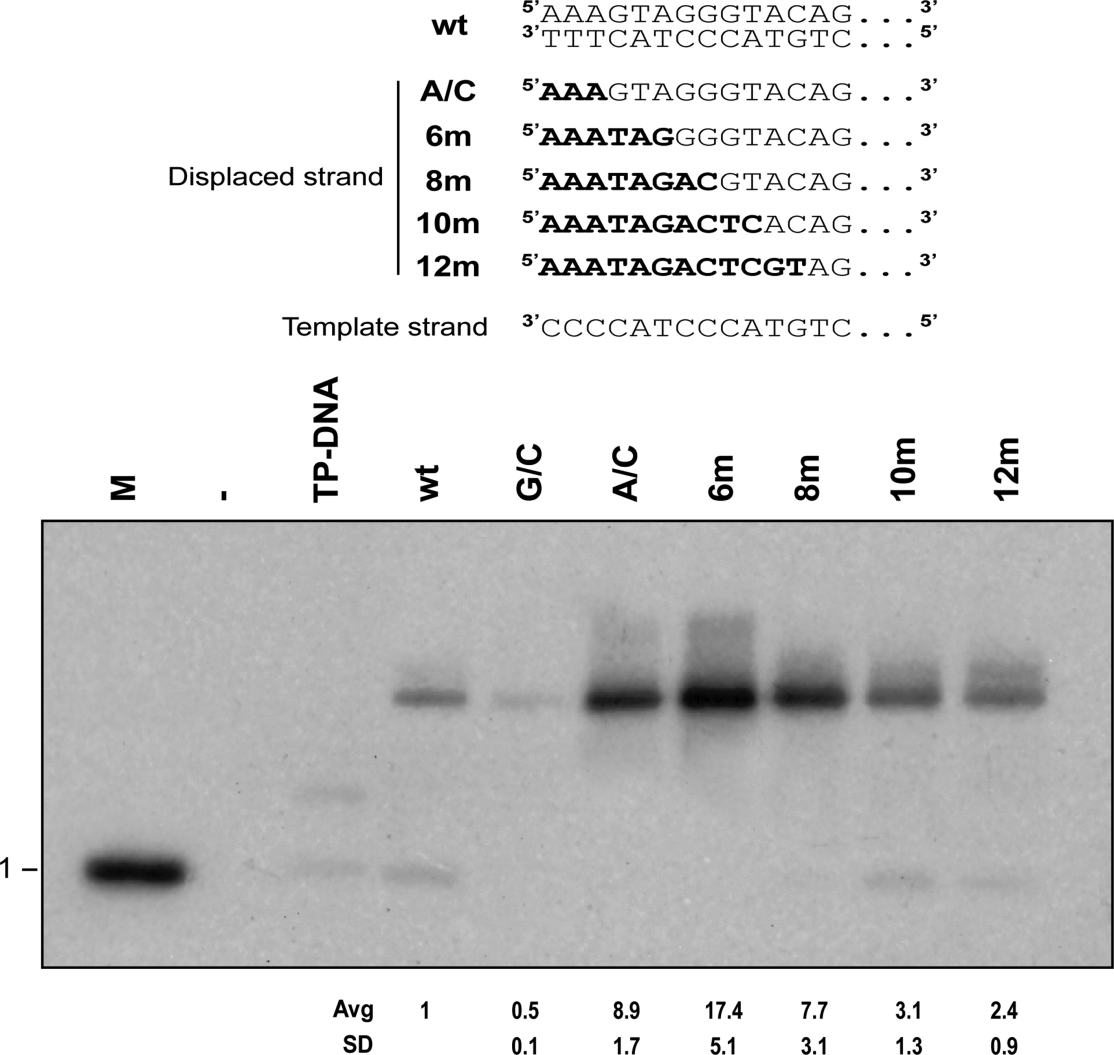
Optimal number of unpaired nucleotides at the replication origin. Notation as before for wt, G/C, A/C and 6m. Number followed by m indicates the number of mismatched positions; these are made taking the 6m construction and substituting progressively the nucleotides 7–12 of the displaced strand (sequence AAATAG^6^GGGTAC^12^) by the non-complementary sequence ACTCGT. Thus, 8m is AAATAGAC, 10 is AAATAGACTC and 12 is AAATAGACTCGT.

Since the artificial origin with six unpaired nucleotides (6m) at the end was the strongest of the combinations tested, we wondered whether longer unpaired regions could have even stronger positive effects on origin utilization. For that purpose, we generated origins with a total of 8, 10 or 12 unpaired nucleotides at the end. To eliminate as much as possible the effect of sequence, we designed origins sharing the template strand sequence and the six first nucleotides of the displaced strand. So, for example, the construction 8m has eight unpairings, of which, the first six were the same as that of construction 6m but the nucleotides at positions 7 and 8 are different on the displaced strand so they generate mismatches. Also, in this experiment, to avoid melting effects due to unpairing of substantial portions of 29mer oligonucleotides, we utilized 68mer oligonucleotides since, for the 68mers, the melting effect of the unpaired portions is negligible at the temperature tested. A control experiment was performed to compare the replication activities of 29mer and 68mer templates (see Supplementary Figure S2). The results (Figure 4) show that the optimum unpairing is six nucleotides since this construct has the highest activity, 17.4-fold that of the wild-type (lane 6m). This maximal activity is lower (about 2-fold) than that obtained with 29mer oligonucleotides, and we presently do not know the reason for this. As further unpairing is added, the activity keeps getting reduced down to 2.4-fold the wild-type activity for 12 mismatched positions (lane 12m).

### Replication steps affected by the unpaired replication origins

**Figure 5.**
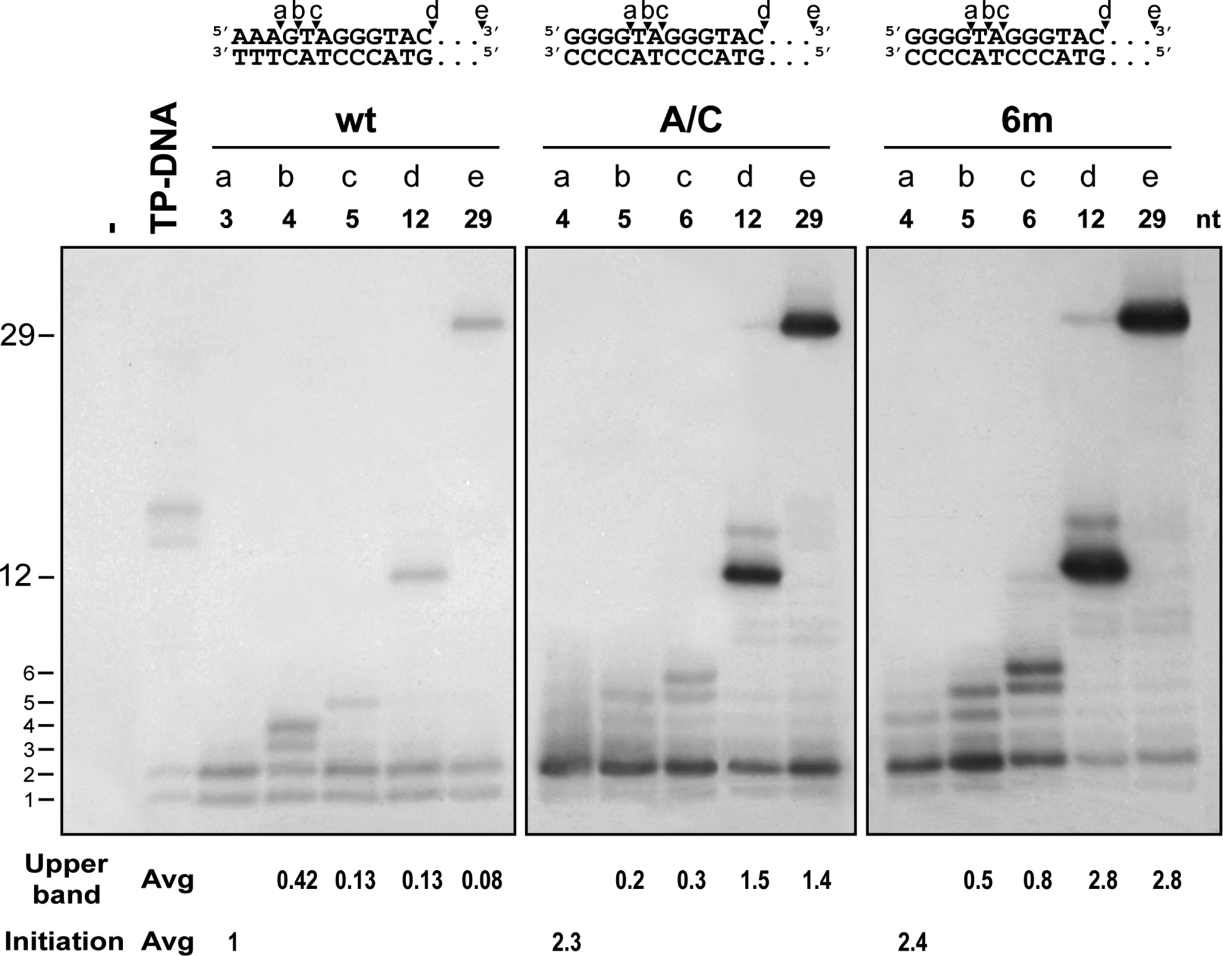
Replication steps affected by the artificial origins. Reactions were prepared as described in Materials and Methods. wt, A/C and 6m origins were assayed in the presence of different nucleotide combinations. Above the groups of lanes we show the sequence of the template strands and its complementary indicating with arrows the products allowed to be synthesized for each lane (see also main text). Numbers indicate the maximum size of DNA for the different products. The reaction products were analyzed by high-resolution SDS-PAGE with 12% acrylamide. The mean values for the quantification of the most relevant bands referred to the wt with the corresponding standard deviations are shown (see Materials and Methods section).

As mentioned in the Introduction, phage Φ29 DNA replication starts with an initiation step, up to the incorporation of the third nucleotide and then follows a transition step until the nascent strand has 6–9 nucleotides. At that point some change takes place and the DNA polymerase switches to the elongation mode from the 10th position onwards after the DNA polymerase and the TP have dissociated. Some of the replication origins described in previous sections were stronger than the wild-type origin, especially those with the AAA/CCC structure at the very end. In order to analyze what step or steps of the replication process are being facilitated in these artificial origins we performed transition assays (see Materials and Methods) with the A/C and 6m origins (Figure 5). In these truncated elongation experiments the reactions included: lanes a, only the first nucleotide to be incorporated (to produce AAA or GGGG depending on the template; initiation bands); lanes b, the first nucleotide (A or G) plus ddG (to yield AAAG in the wild-type origin) or plus ddT (to yield GGGGT in the A/C and 6m origins); lanes c, nucleotides A, G plus ddT (to produce AAAGT in the wild-type origin) or nucleotides G, T plus ddA (to produce GGGGTA in the A/C and 6m origins); lanes d, nucleotides A, G and T plus ddC (that allow polymerization of 12 nucleotides in all constructs) and lanes e, the four nucleotides for full replication of the template. We observed that, for the wild-type origin, the initiation bands (lane a) progressed with some efficiency to position +4 (lane b), but with much less efficiency to positions +5, +12 or full length (lanes c, d, e), taking into account the number of labeled nucleotides that can be incorporated for each length of synthesized DNA. For the A/C and 6m templates we observed an increase in the initiation bands of 2.3 to 2.4 times with respect to those of the wild-type origin but also a further and stronger increase when the replication proceeds to position +12 (11-fold, A/C, or 21-fold, 6m, increase) or full length. The results suggest that the A/C sequences plus the unpairings enhance the initiation step but they also strongly stimulate the passage from transition to elongation.

In this experiment, bands lower than full-length (typically +1 and +2) can arise as final products of abortive initiation or as products cycling in a polymerization-exonucleolysis equilibrium. Both cases imply a difficulty for the polymerase to pass from initiation to transition. For the wild-type origin, looking at the relative amount of +12 or +29 to +1/+2 products it is clear that there is a low overall efficiency in the generation of full-length products. For the A/C and the 6m origins there is a higher initiation but the proportion of initiation to full-length products is clearly much better than for the wild-type origin. This improvement could be achieved by any of the two possibilities, by lowering the proportion of abortive products or by shifting the equilibrium toward polymerization. The simplest explanation for both cases is that the polymerase is not experiencing such a difficulty to do the transition. The fact that in the 6m origin we observe a decrease in the +1/+2 products indicates that a substantial proportion of short products is being extended, which, again, suggests a facilitated transition. As the polymerase molecules transit to elongation they leave the origin free for new heterodimers to initiate replication, and that is probably the explanation for the fact that the amounts of +12 and +29 products obtained with the origins A/C and 6m is higher than expected from the initiation bands.

### Role of the N-terminal domain of the Φ29 TP in replication using artificial origins

**Figure 6.**
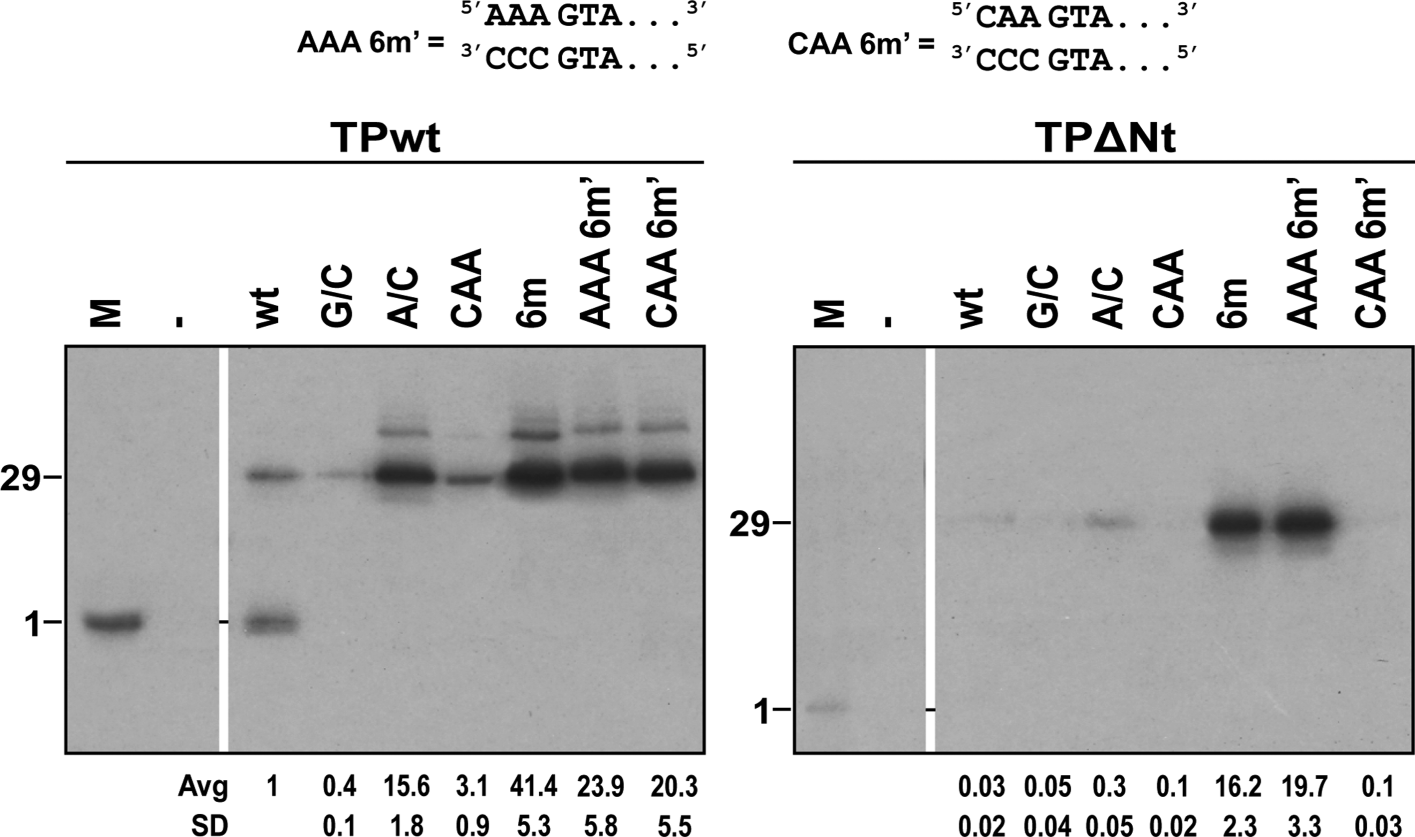
Role of the TP Nt domain in replication using artificial replication origins. Notation of origins as described before. AAA6m’ denotes AAAGTA/CCCGTA and CAA6m’ denotes CAAGTA/CCCGTA. The replication reactions were performed in the presence of 150 nM of either wild-type TP (TPwt) or the ΔNt deletion mutant of TP (TPΔNt). M, marker for the initiation band for the corresponding TP.

It has been reported that the TP N-terminal (Nt) domain has non-sequence specific DNA binding capacity and also that it is partially dispensable for *in vitro* replication of Φ29 DNA (21). To understand the role of the Nt domain in the first steps of replication, we tested a TP mutant that lacks the Nt domain (TPΔNt) in replication assays with different double-stranded origins. As shown before, the identity in the 5′ end of the displaced strand affected the reaction efficiency so we hypothesized that the Nt domain of the TP could have a recognition role of the 5′ end. Thus, we performed replication assays using the TPΔNt and different origins altered on the first 5′ nucleotide. In order to use the same non-template sequences as those in Figure 3, we constructed a six mismatched origin with the sequence AAAGTA/CCCGTA (AAA6m’), and the mutant lacking one A at the 5′ end CAAGTA/CCCGTA (CAA6m’). As shown in Figure 6 (lanes wt), replication utilizing the wild-type origin as template was very much reduced with the TPΔNt, about 30-fold compared to that of the wild-type TP. Similarly, the origins A/C and CAA (both with three mismatches) showed very low utilization (lanes A/C and CAA) in the presence of the TPΔNt. However, the six mismatched constructs showed strong activity in the presence of TPΔNt (lanes 6m and AAA 6m’) and this activity was clearly dependent on A at the 5′ end (lane CAA 6m’). Therefore, origin utilization by the TPΔNt complex has a preference for A at the 5′ position in the six mismatched construct. At the same time, the wild-type TP is hardly affected by the change of the 5′ A into C in the 6m origin (lane CAA 6m’).

From these results we can conclude that: (i) the Nt domain is important for the utilization of paired or three-mismatched origins, (ii) the Nt domain is less important for the utilization of six-mismatched origins with A at the 5′ end of the displaced strand and (iii) the Nt domain is not required for the preference for A at the 5′ end of the displaced strand by the TP/DNA polymerase complex. These results agree with the available evidence that suggests that the Nt domain is required for efficient binding to double-stranded DNA, without sequence specificity (33). The results also imply that the other domains of the TP (I, Ct) and/or regions of the Φ29 DNA polymerase would be responsible for the 5′ A preference.

### Effect of protein p6 on the artificial replication origins

**Figure 7.**
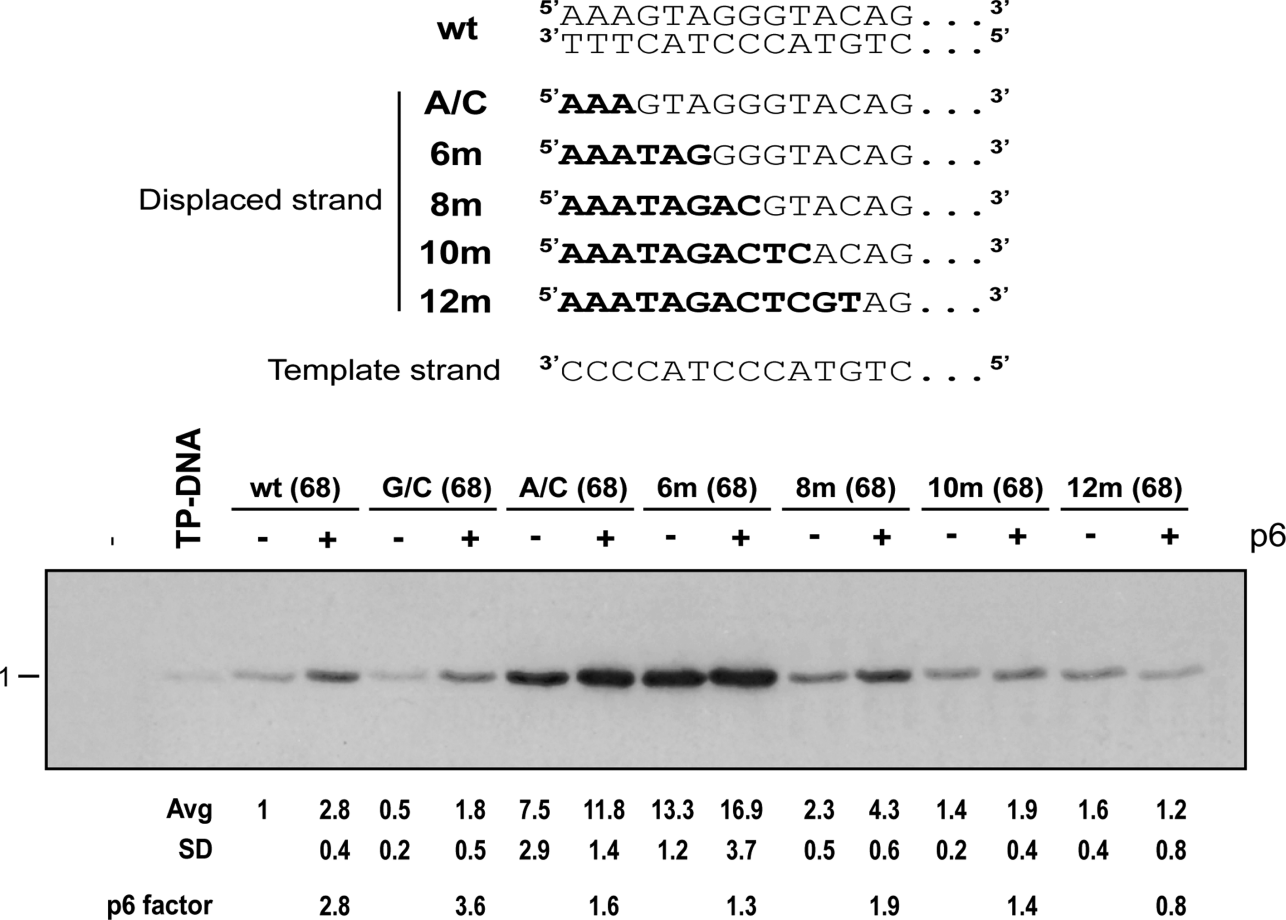
Effect of p6 on the artificial replication origins. Initiation reactions were performed as described (see Materials and Methods section) but having an initial nucleotide concentration of 0.1 μM. The double-stranded DNA origins were constructed with oligonucleotides 68-nucleotides long, containing a p6 nucleation site, but the relevant ends were the same as those in the corresponding 29 bp origins. Where indicated, protein p6 was included in the reaction at a concentration of 35 μM. Quantification of the initiation bands are normalized in relation to the wt band (wt = 1); the corresponding standard deviations are shown. Stimulation factors by p6 are indicated for each origin.

As mentioned in the Introduction, two non-excluding mechanisms have been proposed to explain the p6 role in the stimulation of replication: to melt the DNA strands at the very ends of the phage genome and to recruit the TP/DNA polymerase preinitiation complex to the origin of replication. Given that some of the artificial origins described here have their ends already unpaired we considered they could serve as a test to unravel the p6 mechanism. The effect of p6 is mostly evident in the initiation step with a moderately low nucleotide concentration (0.1 μM), so we performed an initiation assay (see Materials and Methods section) using 68 bp double-stranded origins containing the high affinity p6 nucleation sites localized between positions 35 and 58 (17), and the most relevant end sequences and structures described in this study. In Figure 7, the p6-induced factors of stimulation were 2.8 for the wt origin, 3.6 for GC and 1.6, 1.3, 1.9, 1.4 and 0.8 for the A/C, 6m, 8m, 10m and 12m origins, respectively. It is observed that the effect of p6 is lower with the unpaired origins down to no effect with 12 mismatches. This lower level of stimulation by p6 would suggest that, at least in part, the unpaired origins are favoring the same step of replication as p6. And this in turn is in agreement with the explanation that the stimulation by p6 and the effects of the unpaired origins act through the melting of the dsDNA at the origin. Notwithstanding, an alternative explanation would be that the unpairings would dissipate the torsional strength generated by the p6 complex therefore affecting its function. In any case, with the exception of the 12m origin, there is always a remaining level of stimulation by p6 that could be attributed to a different p6 action-mechanism like the direct interaction of p6 with the complex TP/DNA polymerase, as it has been previously suggested (20,21).

## DISCUSSION

This study has helped to deepen our understanding of the first steps in the protein-primed DNA replication of bacteriophage Φ29. Previous studies showed that the first three nucleotides of the template strand were important for the sliding-back mechanism and also the identity of some of the base pairs from positions 4–12 could affect the activity of the replication origin. In this study, we have observed that *in vitro* active origins can be built with different triplet combinations at the ends and even forming unpaired ends. We have determined that the preferred triplet at the template strand is CCC. Even when CCC is not the wild-type sequence, this preference could be due to the strong pairing G/C that would stabilize the complex TP/DNA polymerase + CCC template + first incorporated nucleotides (Gs), in a way that more initiations could be produced and, from those, a higher proportion could progress to elongation. After the C, the other preferred template is the wt (TTT), constituted by the other pyrimidine, T, and after those, at a distance, the next are the two purines, A and G, probably because the initiation complex does not accommodate well the volume of the purines in the template strand.

In the displaced strand there is a preference for AAA at the 5′ end when there are Cs at the corresponding positions of the template strand. Active origins can also be constructed with Gs, Ts or Cs at the 5′ end of the displaced strand but their activity as origins depends on the nucleotides at the template strand. The low activity of the G/C paired origin is probably because it requires more energy for melting as compared to the wt A/T origin or any unpaired origin. The unpairing of the origin seems to have a positive effect but it has to be taken into account the nucleotide sequences at the template and at the displaced strands to predict the strength of an origin. Thus, there appears to be a mutual influence of these three parameters: template sequence, displaced strand sequence and unpairing. To this we have to add the effect of the identity of nucleotides at positions 4–6 and the unpairing at these positions. For paired ends, we have observed that the effect of the identity at positions 4–6 is little and, in most cases, a change from the wt sequence has a negative effect. On the other hand, the unpairing causes an increase up to 10-fold on the origin activity. Also, interestingly, the effect of this unpairing is additive with the effect of A/C on origin utilization. Thus, the A/C unpaired end gives an origin activity commonly around 15-fold higher than that of the wt, and this effect would add up with that of the internal unpairing, about 10-fold, to yield an activity for the 6m of more than 25-fold respect to that of the wt. The fact that this effect is approximately additive would imply that the initiation step/s facilitated by these origins are the same. In the context of the six nucleotides unpairing, the sequence effects are relatively small, including the presence of AAA at the 5′ end; this could be explained assuming that for these constructs the largest effect is due to the unpairing. Altogether the results suggest that the initial steps of the replication are critical and there are some barriers for the wt origin that can be overcome: (i) by stabilizing the initiation complex if the first nucleotides incorporated have stronger (G/C) pairings with the template, (ii) by unwinding parts of the origin and (iii) by having AAA at the 5′ end.

The AAA preference at the displaced strand requires a recognizable origin structure even when partially unpaired because, if we add 10 nucleotides to the displaced strand there is almost no positive effect of the AAA sequence whether at the very end or opposed to the template. To explain this, a possibility would be that a non-template strand of certain length would tend to fit the displaced strand path that probably exists on the surface of the elongating DNA polymerase, and this would preclude the AAA end-recognition that would occur only at the initiating conformation of the polymerase. The preference for AAA at the 5′ end of the origin can be considered an additional fidelity mechanism because the AAA at the 5′ end requires that the template strand is TTT, this is, the correct sequence that is required for the sliding-back mechanism. An incorrect sequence at the 3′ end will not have AAA at 5′ and therefore would be recognized with much lower efficiency by the TP/DNA polymerase heterodimer. It is also noteworthy the fact that a 5′ recessive end or a 5′ hairpin have very low activity and this underlines the importance of the presence of a correct displaced strand in the initiation of replication. The results also show that the sequence-independent DNA binding domain of the TP, the Nt domain, would not be required for the recognition of the 5′ A. However, the Nt domain does appear to be critical for double-stranded or even three mismatched origin utilization by the TP/DNA polymerase heterodimer, and, reciprocally, the effect of the Nt domain becomes less critical in origins with more unpaired sequences. This also suggests that the preference of the TP/DNA polymerase for an unpaired origin does not require the Nt domain. Also, it is worth noting that, while the optimal unpairing for the origin is six nucleotides, longer unpairings still constitute active origins.

Regarding the specific step of the Φ29 TP-primed replication process that is stimulated by the artificial origins, there is a clear stimulation of the initiation step probably because the unpairing and C sequences stabilize the initial recruitment of the TP/DNA polymerase heterodimer. However, there is a stronger stimulation of the transition-to-elongation passage, and this could be due to the alleviation of the thermodynamic barrier that poses the melting of the dsDNA at the point where the interactions between TP and DNA polymerase are being broken (31).

Our data show parallelism between the effect of the artificial origins and the p6-induced stimulation of initiation. Protein p6 has been proposed to act on the unwinding of the origins, and, in agreement with that, we describe here origins with partially unpaired ends showing increased utilization. Interestingly, these origins show a low stimulation by p6. The simplest explanation for this lower stimulation is that the artificial origins and p6 stimulate, at least in part, the same step of the replication process. There is a second mechanism proposed for p6 that is the recruitment of the heterodimer TP/DNA polymerase by means of protein–protein contacts with p6 proteins bound to the origins. This mechanism could still be acting in the artificial DNA origins, taking into account there is still a certain level of stimulation by p6 in some origins.

Some of the findings in this study show similarities with Adenovirus and phage PRD1 DNA replication. For example, it has been shown that the heterodimer Ad pTP/DNA polymerase recognizes a sequence located at positions 8–17 from the origin end, within the ITR (38), and also the PRD1 complex requires the terminal 20 bp of the ITR, this sequence being more active as dsDNA than as ssDNA (39). For the Φ29 TP/DNA polymerase complex there is also some sequence preference for the origin, including the ITR, but that preference is less clear than for Adeno or PRD1, and, in any case, in Φ29 there is indeed a clear preference for dsDNA and even more so for unpaired ends. Another similarity between Adeno and Φ29 DNA replication origins is the fact that both are stimulated by additional DNA binding proteins to obtain efficient replication: DBP and cellular factors NF-I and NF-III for Adeno (40) and p6 for Φ29, and all these proteins bind dsDNA near the origin ends, and indeed stimulation by Ad DBP requires a dsDNA origin.

**Figure 8.**
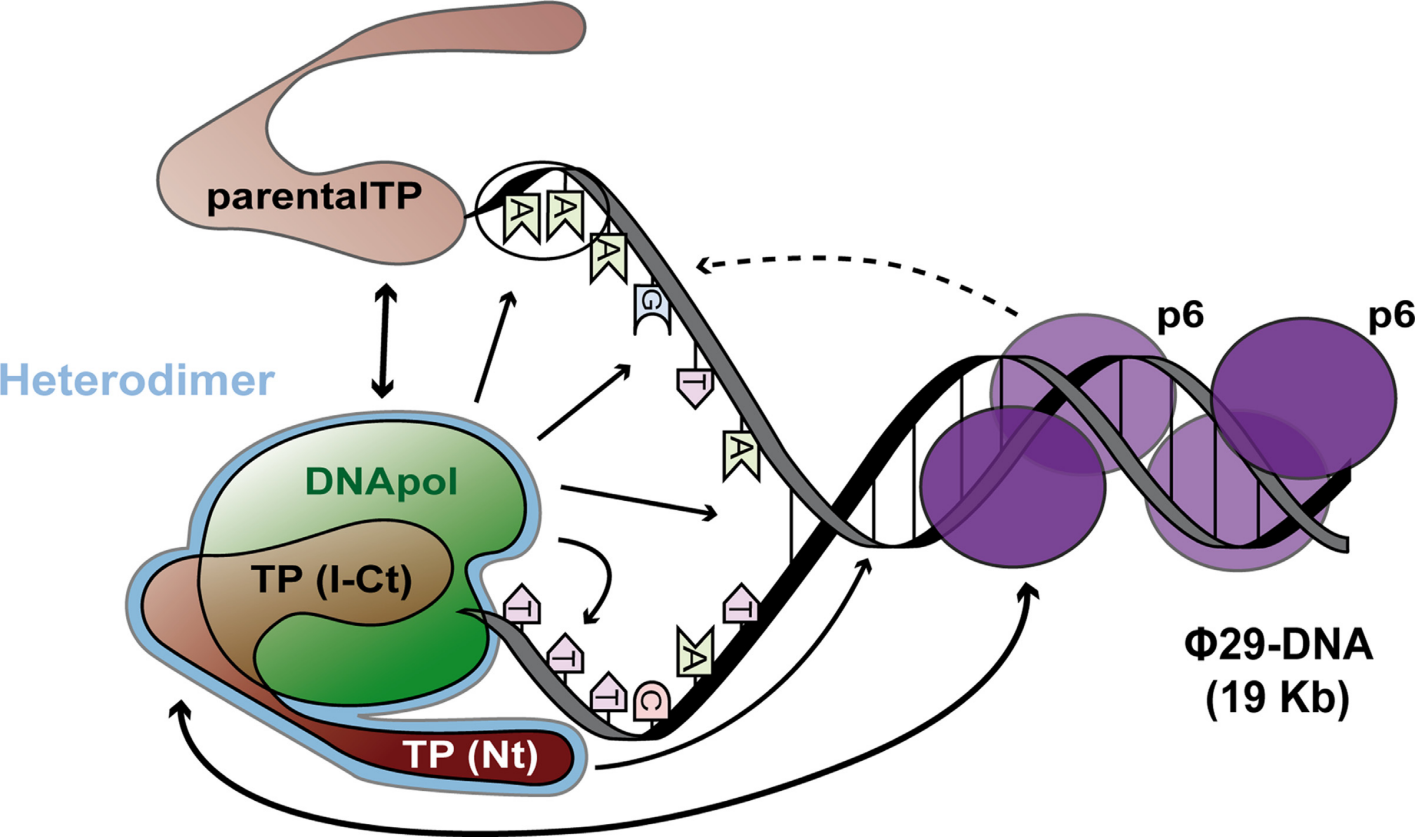
Model of phage Φ29 DNA initiation of replication interactions. DNA polymerase (DNA pol), parental TP, TP domains I to Ct (TP (I-Ct)), TP domain Nt (Nt) and p6 (p6) are shown in schematic form. Single-arrowed lines denote protein-DNA interactions, double-arrowed lines denote protein–protein interactions, and the discontinuous line indicates the unwinding effect of p6 on the DNA. The six first nucleotides of the template and displaced strands are indicated.

A model that takes into account the results presented in this article, together with previous information, is shown in Figure 8. In slow motion, a possible order of events for Φ29 early replication would be as follows. The parental TP and the p6 complex bound at the end of the Φ29 genome would recruit the TP/DNA polymerase heterodimer to the origin. Then, the unwinding of the origin would start, possibly helped by protein p6, and the TP/DNA polymerase complex would bind to the template strand and recognize the AAA at the 5′ end of the displaced strand, and this complex probably also interacts with sequences from the fourth bp on. At the same time, the Nt domain would stabilize the complex by binding to dsDNA regions of the origin in a non-sequence specific fashion, and the initiating nucleotides would start being incorporated. Further unwinding of the origin would favor the binding of the TP/DNA polymerase complex to the displaced strand and the continuation of replication through the transition phase. At that point a thermodynamic barrier would have to be passed by continuing the unwinding of the DNA strands at the same time as it happens the dissociation of the DNA polymerase from the TP utilized as primer. These events are probably driven by the nascent DNA chain. After the passage from transition to elongation, the DNA polymerase, now dissociated from the TP, would continue in a processive, strand-displacing, mode to replicate the whole phage genome.

The results presented here, not only help to understand the early events at a TP-containing replication origin, but also they serve as basis to design more efficient TP-primed replication procedures. Heterologous DNAs placed between Φ29 DNA replication origins do not have the parental TP covalently bound to the 5′ ends and we have observed that the efficiency of amplification of those DNAs is moderate, measuring efficiency as number of initial template molecules required to obtain amplification. To overcome this problem we plan to incorporate some of the origins described here into the design of new replication vectors that could compensate for the absence of parental TP. The goal for such vectors would be to achieve strong amplification starting from a low number of template molecules. As mentioned in the Introduction, this would facilitate a number of applications based on the efficient production of defined protein-DNA covalent amplicons.

## SUPPLEMENTARY DATA

Supplementary Data are available at NAR Online.

SUPPLEMENTARY DATA
