# Supplementary material for: Improved artificial origins for phage Φ29 terminal protein-primed replication. Insights into early replication events
Source: Nucleic Acids Res. 2014 Jul 31;42(15):9792–806. doi: 10.1093/nar/gku660 (PMC4150772; doi:10.1093/nar/gku660)
Supplement: SUPPLEMENTARY DATA [file supp_42_15_9792__index.html]

Improved artificial origins for phage Φ29 terminal protein-primed replication. Insights into early replication events — SUPPLEMENTARY DATA 

# Improved artificial origins for phage Φ29 terminal protein-primed replication. Insights into early replication events

## SUPPLEMENTARY DATA

**Files in this Data Supplement:**

- SUPPLEMENTARY DATA
