# Supplementary material for: Improved artificial origins for phage Φ29 terminal protein-primed replication. Insights into early replication events
Source: Nucleic Acids Res. 2014 Jul 31;42(15):9792–806. doi: 10.1093/nar/gku660 (PMC4150772; doi:10.1093/nar/gku660)
Supplement: SUPPLEMENTARY DATA [file supp_gku660_nar-00928-v-2014-File011.pdf]

	Template	Sequence
	wt	5'AAAGTAGGGTACAGCGACAACATACACCA3'<br>3'TTTCATCCCATGTCGCTGTTGTATGTGGT5'
	G/T	5'**GGG**GTAGGGTACAGCGACAACATACACCA3'<br>3'**TTT**CATCCCATGTCGCTGTTGTATGTGGT5'
	T/T	5'**TTT**GTAGGGTACAGCGACAACATACACCA3'<br>3'**TTT**CATCCCATGTCGCTGTTGTATGTGGT5'
	C/T	5'**CCC**GTAGGGTACAGCGACAACATACACCA3'<br>3'**TTT**CATCCCATGTCGCTGTTGTATGTGGT5'
	A/C	5'**AAA**GTAGGGTACAGCGACAACATACACCA3'<br>3'**CCC**CATCCCATGTCGCTGTTGTATGTGGT5'
	G/C	5'GGGGTAGGGTACAGCGACAACATACACCA3'<br>3'CCCCATCCCATGTCGCTGTTGTATGTGGT5'
	T/C	5'**TTT**GTAGGGTACAGCGACAACATACACCA3'<br>3'**CCC**CATCCCATGTCGCTGTTGTATGTGGT5'
	C/C	5'**CCC**GTAGGGTACAGCGACAACATACACCA3'<br>3'**CCC**CATCCCATGTCGCTGTTGTATGTGGT5'
	A/A	5'**AAA**GTAGGGTACAGCGACAACATACACCA3'<br>3'**AAA**CATCCCATGTCGCTGTTGTATGTGGT5'
	G/A	5'**GGG**GTAGGGTACAGCGACAACATACACCA3'<br>3'**AAA**CATCCCATGTCGCTGTTGTATGTGGT5'
	T/A	5'TTTGTAGGGTACAGCGACAACATACACCA3'<br>3'AAACATCCCATGTCGCTGTTGTATGTGGT5'
	C/A	5'**CCC**GTAGGGTACAGCGACAACATACACCA3'<br>3'**AAA**CATCCCATGTCGCTGTTGTATGTGGT5'
	A/G	5'**AAA**GTAGGGTACAGCGACAACATACACCA3'<br>3'**GGG**CATCCCATGTCGCTGTTGTATGTGGT5'
	G/G	5'**GGG**GTAGGGTACAGCGACAACATACACCA3'<br>3'**GGG**CATCCCATGTCGCTGTTGTATGTGGT5'
	T/G	5'**TTT**GTAGGGTACAGCGACAACATACACCA3'<br>3'**GGG**CATCCCATGTCGCTGTTGTATGTGGT5'
	C/G	5'CCCGTAGGGTACAGCGACAACATACACCA3'<br>3'GGGCATCCCATGTCGCTGTTGTATGTGGT5'
	wt_L	5'AAAGTAAGCCCCCACCCTCACATGATACC3'<br>3'TTTCATTCGGGGGTGGGAGTGTACTATGG5'
	wt_{SS}	3'TTTCATCCCATGTCGCTGTTGTATGTGGT5'
	$GA\text{-}1_L$	5'AAATAGAGTCCACACCCTCACATGATACC3'<br>3'TTTATCTCAGGTGTGGGAGTGTACTATGG5'
	A/T^{TAG}	5'AAATAGGGGTACAGCGACAACATACACCA3'<br>3'TTTATCCCCATGTCGCTGTTGTATGTGGT5'
	A/T^{TAG}_{CAT}	5'AAA**TAG**GGGTACAGCGACAACATACACCA3'<br>3'TTT**CAT**CCCATGTCGCTGTTGTATGTGGT5'
	A/T^{GTA}_{ATC}	5'AAA**GTA**GGGTACAGCGACAACATACACCA3'<br>3'TTT**ATC**CCCATGTCGCTGTTGTATGTGGT5'
	A/T^{TCA}	5'AAATCAGGGTACAGCGACAACATACACCA3'<br>3'TTTAGTCCCATGTCGCTGTTGTATGTGGT5'
	A/T^{AGT}	5'AAAAGTGGGTACAGCGACAACATACACCA3'<br>3'TTTTCACCCATGTCGCTGTTGTATGTGGT5'
	A/T^{CGA}	5'AAACGAGGGTACAGCGACAACATACACCA3'<br>3'TTTGCTCCCATGTCGCTGTTGTATGTGGT5'
	A/T^{CAT}	5'AAACATGGGTACAGCGACAACATACACCA3'<br>3'TTTGTACCCATGTCGCTGTTGTATGTGGT5'
	G/C^{TAG}_{CAT}	5'GGG**TAG**GGGTACAGCGACAACATACACCA3'<br>3'CCC**CAT**CCCATGTCGCTGTTGTATGTGGT5'
	(6m) A/C^{TAG}_{CAT}	5'**AAATAG**GGGTACAGCGACAACATACACCA3'<br>3'**CCCCAT**CCCATGTCGCTGTTGTATGTGGT5'
	A/ddC	5'**AAA**GTAGGGTACAGCGACAACATACACCA3'<br>3'**ddCCC**CATCCCATGTCGCTGTTGTATGTGGT5'
	G/ddC	5'**GGG**GTAGGGTACAGCGACAACATACACCA3'<br>3'**ddCCC**CATCCCATGTCGCTGTTGTATGTGGT5'
	10+A/ddC	5'ATCACTATGC**AAA**GTAGGGTACAGCGACAACATACACCA3'<br>3'**ddCCC**CATCCCATGTCGCTGTTGTATGTGGT5'
	10+G/ddC	5'ATCACTATGCGGGGTAGGGTACAGCGACAACATACACCA3'<br>3'ddCCCCATCCCATGTCGCTGTTGTATGTGGT5'
	10+TAC/ddC	5'ATCACTATGC**TAC**GTAGGGTACAGCGACAACATACACCA3'<br>3'**ddCCC**CATCCCATGTCGCTGTTGTATGTGGT5'
	AAA10	5'AAAACTATGC**TAC**GTAGGGTACAGCGACAACATACACCA3'<br>3'**ddCCC**CATCCCATGTCGCTGTTGTATGTGGT5'
	5'hA/T	5'CGCTATCTTAGCGAAAGTAGGGTACAGCGACAACATACACCA3'<br>3'TTTCATCCCATGTCGCTGTTGTATGTGGT5'
	5'Δ6wt	5'GGGTACAGCGACAACATACACCA3'<br>3'TTTCATCCCATGTCGCTGTTGTATGTGGT5'
	AAC	5'**AAC**GTAGGGTACAGCGACAACATACACCA3'<br>3'**CCC**CATCCCATGTCGCTGTTGTATGTGGT5'
	ACA	5'**ACA**GTAGGGTACAGCGACAACATACACCA3'<br>3'**CCC**CATCCCATGTCGCTGTTGTATGTGGT5'
	CAA	5'**CAA**GTAGGGTACAGCGACAACATACACCA3'<br>3'**CCC**CATCCCATGTCGCTGTTGTATGTGGT5'
	ACC	5'**ACC**GTAGGGTACAGCGACAACATACACCA3'<br>3'**CCC**CATCCCATGTCGCTGTTGTATGTGGT5'
	CAC	5'**CAC**GTAGGGTACAGCGACAACATACACCA3'<br>3'**CCC**CATCCCATGTCGCTGTTGTATGTGGT5'
	CCA	5'**CCA**GTAGGGTACAGCGACAACATACACCA3'<br>3'**CCC**CATCCCATGTCGCTGTTGTATGTGGT5'
	CCC	5'**CCC**GTAGGGTACAGCGACAACATACACCA3'<br>3'**CCC**CATCCCATGTCGCTGTTGTATGTGGT5'
	AAA 6m'	5'**AAAGTA**GGGTACAGCGACAACATACACCA3'<br>3'**CCCGTA**CCCATGTCGCTGTTGTATGTGGT5'
	CAA 6m'	5'**CAAGTA**GGGTACAGCGACAACATACACCA3'<br>3'**CCCGTA**CCCATGTCGCTGTTGTATGTGGT5'
68-mer	wt	5'AAAGTAGGGTACAGCGACAACATACACCATTTCCCCATTGACCGACTATCTTCGACAAGAATCTAACA3'<br>3'TTTCATCCCATGTCGCTGTTGTATGTGGTAAAGGGGTAACTGGCTGATAGAAGCTGTTCTTAGATTGT5'
68-mer	A/C	5'**AAA**GTAGGGTACAGCGACAACATACACCATTTCCCCATTGACCGACTATCTTCGACAAGAATCTAACA3'<br>3'**CCC**CATCCCATGTCGCTGTTGTATGTGGTAAAGGGGTAACTGGCTGATAGAAGCTGTTCTTAGATTGT5'
68-mer	6m	5'**AAATAG**GGGTACAGCGACAACATACACCATTTCCCCATTGACCGACTATCTTCGACAAGAATCTAACA3'<br>3'**CCCCAT**CCCATGTCGCTGTTGTATGTGGTAAAGGGGTAACTGGCTGATAGAAGCTGTTCTTAGATTGT5'
68-mer	8m	5'**AAATAGAC**GTACAGCGACAACATACACCATTTCCCCATTGACCGACTATCTTCGACAAGAATCTAACA3'<br>3'**CCCCATCC**CATGTCGCTGTTGTATGTGGTAAAGGGGTAACTGGCTGATAGAAGCTGTTCTTAGATTGT5'
68-mer	10m	5'**AAATAGACTC**ACAGCGACAACATACACCATTTCCCCATTGACCGACTATCTTCGACAAGAATCTAACA3'<br>3'**CCCCATCCCA**TGTCGCTGTTGTATGTGGTAAAGGGGTAACTGGCTGATAGAAGCTGTTCTTAGATTGT5'
68-mer	12m	5'**AAATAGACTCGT**AGCGACAACATACACCATTTCCCCATTGACCGACTATCTTCGACAAGAATCTAACA3'<br>3'**CCCCATCCCATG**TCGCTGTTGTATGTGGTAAAGGGGTAACTGGCTGATAGAAGCTGTTCTTAGATTGT5'
Marker	15	3'TTTCATCCCATGTCG5'
Marker	17	3'TTTCATCCCATGTCGCT5'
Marker	19	3'TTTCATCCCATGTCGCTGT5'
Marker	21	3'TTTCATCCCATGTCGCTGTTG5'
Marker	23	3'TTTCATCCCATGTCGCTGTTGTA5'
Marker	25	3'TTTCATCCCATGTCGCTGTTGTATG5'
Marker	27	3'TTTCATCCCATGTCGCTGTTGTATGTG5'

Supplementary Table I. List of oligonucleotides and hybridized origins used in this article. Boldface refers to the mismatched positions at the DNA template.

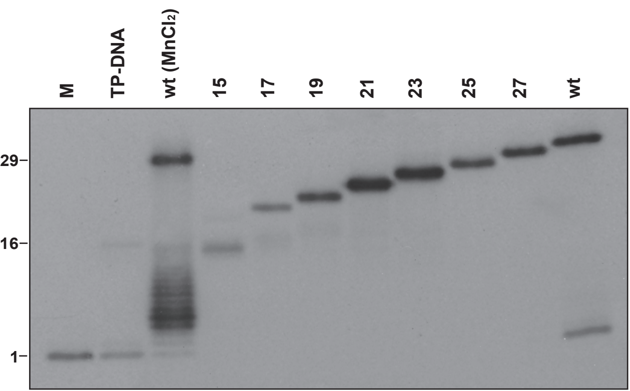

Supplementary Figure 1. Molecular weight ladder for 29mer-origin replication reactions. M, initiation reaction marker (reaction using TP-DNA as template and dATP as the only nucleotide); TP-DNA, control replication reaction with the TP-containing Φ29 genome as template (initiation [+1] and a partial elongation band are produced [+16]); wt (MnCl2), replication reaction using as template the wt Φ29 DNA right end 29mer origin with Mn2+ as metal cofactor; 15 to 27, replication reactions performed using as template the wt right origin single-stranded oligonucleotides of the specified length, counting from the right extreme (see Supplementary table 1 and Methods); wt, replication reaction using as template the wt Φ29 DNA right end 29mer origin. Replication conditions for wt were the standard ones used in this article, this is, in the presence of Mg^{2+} ($MgCl_2$) as metal cofactor (see replication assay in Methods for the rest of the conditions). The use of Mn^{2+} ($MnCl_2$) as cofactor for the reaction wt produces a series of partial elongation bands that allow counting positions from +1 to +12 nucleotides added to the TP. Replication conditions for templates 15mer to 27mer were the same as that for the double-stranded wt 29mer origin, except that the concentration of the single-stranded templates was 626 nM.

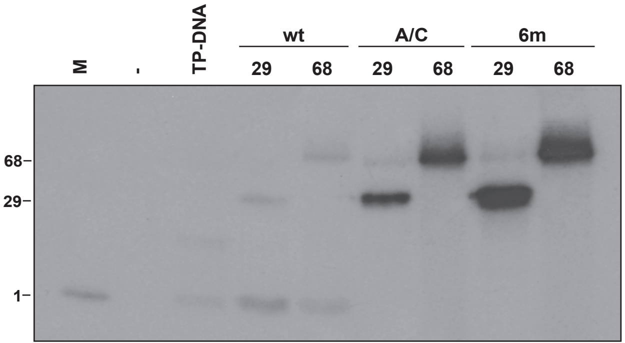

	wt 29	wt 68	A/C 29	A/C 68	6m 29	6m 68
Avg	1	1.1	12.5	8.7	45.8	15.9
SD		0.2	2	1.8	4.4	4.2

Supplementary Figure 2. Replication assay with 29 and 68mer versions of the origins wt, A/C and 6m. Double-stranded DNA origins were assembled as explained in the main text and the replication conditions were the standard ones used in this article (see replication assay in Methods). The positions corresponding to TP-dAMP (1) and full lenght products, TP-29 nucleotides and TP-68 nucleotides as ssDNA are indicated. Lanes are labeled as in the main text. The number of labeled nucleotides incorporated per replicated DNA molecule was taken into account for all the quantifications (68mer templates incorporate from 1.9 to 2.2 more dAMP molecules than the 29mer templates, depending on the origin). At the bottom of the figure are shown the mean values of the quantification of the intensities of the bands corresponding to full lenght products normalized to the wt 29mer (wt = 1), with their corresponding standard deviations. Values are the average of at least three independent experiments.
